# Cardiovascular Risk in Psoriatic Arthritis: Mechanisms, Risk Assessment, and Long-Term Management Implications

**DOI:** 10.3390/ijms27104226

**Published:** 2026-05-09

**Authors:** Stefan Totolici, Ana-Maria Vrabie, Catalin Adrian Buzea, Elisabeta Badila

**Affiliations:** 1 Cardiology Department, Carol Davila University of Medicine and Pharmacy, 050474 Bucharest, Romania; ana-maria.vrabie@drd.umfcd.ro (A.-M.V.); catalin.buzea@umfcd.ro (C.A.B.); elisabeta.badila@umfcd.ro (E.B.); 2 Colentina Clinical Hospital, 020125 Bucharest, Romania

**Keywords:** psoriatic arthritis, systemic inflammation, atherosclerosis, cardiovascular risk, cardiovascular disease, major adverse cardiovascular events, cardio-rheumatology

## Abstract

Beyond its typical synovio-entheseal manifestations, psoriatic arthritis (PsA) is a systemic immune-mediated disease that carries a substantial, independent risk of major adverse cardiovascular events (MACE). The complex interaction of chronic systemic inflammation, a high prevalence of traditional risk factors, and PsA treatment drugs results in this increased cardiovascular burden, which is commonly underestimated in cardiology practice. Adipokine balance, insulin signalling, and lipid metabolism are all impacted by cytokine-driven systemic inflammation, which promotes metabolic abnormalities and accelerated atherogenesis. PsA-specific therapy has a complex and significant effect on cardiovascular risk. While there is evidence that strong inflammation suppression with tumour necrosis factor-α (TNF-α) inhibitors may reduce cardiovascular risk, some medications, such as Janus kinase (JAK) inhibitors, should be carefully considered due to potential side effects. In order to outline the epidemiology and pathophysiology of the PsA-cardiovascular risk nexus, this narrative review synthesises recent data. It also offers a critical framework for managing cardiovascular risk in this susceptible group, advocating for a multidisciplinary approach that incorporates strict management of both inflammation and traditional risk factors to lessen the excessive burden of MACE.

## 1. Introduction

Psoriatic arthritis (PsA) is a chronic, immune-mediated inflammatory condition with a variety of extra-articular and musculoskeletal manifestations [1]. PsA was once thought to be primarily a localised rheumatologic condition, but it is now becoming more widely acknowledged as a systemic disorder with far-reaching effects that extend well beyond the joints. Among these, cardiovascular disease (CVD) has become a significant determinant in long-term prognosis, significantly contributing to increased morbidity and premature mortality in this patient population [2]. Epidemiological research over the last 20 years has repeatedly shown that individuals with PsA have a higher incidence of major adverse cardiovascular events (MACE) than the general population [3,4,5]. The higher prevalence of traditional cardiovascular risk factors, including smoking, insulin resistance, dyslipidaemia, obesity, and hypertension, which often cluster in PsA patients, only partially explains this elevated risk. Importantly, even after adjusting for these traditional factors, cardiovascular risk is still elevated, highlighting the role of disease-specific mechanisms inherent to PsA [3,4,6].

One important pathogenic link between PsA and accelerated CVD is chronic systemic inflammation. Premature atherosclerosis is caused by endothelial dysfunction, vascular inflammation, and metabolic abnormalities, all of which are promoted by persistent immune activation. A pro-atherogenic and metabolically detrimental milieu is produced by pro-inflammatory cytokines implicated in PsA pathogenesis, which have pleiotropic effects on oxidative stress, insulin signalling, lipid metabolism, and adipose tissue function [7]. Over time, these inflammatory processes increase cardiovascular vulnerability through dynamic interactions with traditional risk factors [8].

Simultaneously, the cardiovascular implications of PsA therapies have drawn more attention. Pharmacologic agents may have varying effects on cardiovascular risk, even though effective systemic inflammation suppression may reduce it. Therefore, depending on the therapeutic approach used, PsA treatment may either increase or reduce cardiovascular risk [9,10]. Making well-informed clinical decisions requires an understanding of this dual role.

Despite growing awareness of the PsA–CVD connection, cardiovascular risk assessment and prevention remain suboptimal in routine clinical practice. Cardiovascular risk is frequently underestimated, screening is inconsistently applied, and management strategies are often fragmented between specialties [6,11]. These gaps highlight the need for an integrated approach that combines rigorous control of inflammatory disease activity with systematic identification and management of cardiovascular risk factors.

In this context, the present narrative review aims to synthesize contemporary evidence on the epidemiology, pathophysiological mechanisms, and therapeutic determinants of CVD in PsA. Although the association between PsA and CVD has been widely documented, the available literature often addresses individual aspects of this relationship—such as inflammatory mechanisms, cardiometabolic comorbidities, or therapeutic implications—separately. The purpose of this review is therefore not to reiterate established concepts, but to provide an integrated and clinically oriented synthesis that connects three domains that are frequently discussed in isolation: inflammatory and metabolic mechanisms that drive cardiovascular risk in PsA, the cardiovascular impact of contemporary PsA therapies, and practical approaches to cardiovascular risk assessment and integrated management within an emerging cardio-rheumatology framework. By bringing these perspectives together, the review aims to provide a coherent conceptual overview and a clinically relevant framework for cardiovascular risk stratification and long-term management in patients with PsA.

## 2. Epidemiology of Cardiovascular Risk in PsA

Throughout the course of the disease, over half of patients with PsA will experience a wide range of comorbid conditions [12]. The systemic nature of PsA is highlighted by the fact that cardiovascular risk factors and established CVD are among the most common comorbidities. This population has a higher cardiovascular burden, according to epidemiological data. According to Polachek et al.’s meta-analysis, PsA patients had a 55% higher overall cardiovascular risk and a 43% higher incidence of CVD than the general population. Analysing individual cardiovascular outcomes showed significantly increased risks of heart failure, cerebrovascular disease, and myocardial infarction, rising by 68%, 22%, and 31%, respectively [13]. Large population-based data, such as a 2019 study that found a 29% excess risk of CVD among those with PsA, support these findings [14]. Alongside cardiovascular morbidity, there is an elevated risk of mortality. With a relative risk (RR) of 1.74, individuals with PsA have a significantly greater overall mortality rate, according to a nationwide cohort study. The main cause of this increased mortality risk is CVD, for which the RR was 1.84 [15]. In line with these findings, analyses from the Swedish national registry by Juneblad et al. demonstrated a significantly higher cardiovascular mortality rate in PsA compared with the general population [16]. Importantly, even after adjusting for traditional cardiovascular risk factors, this excess cardiovascular risk remains, suggesting that PsA-related mechanisms play a separate role. Furthermore, several studies have demonstrated that cardiovascular risk in PsA is not constant but rather rises with increased disease activity, duration, and cumulative inflammatory burden [7].

Subclinical cardiovascular involvement in PsA is increasingly evident across complementary imaging and functional domains, supporting the concept that vascular disease often precedes clinically overt events. Carotid ultrasound-based studies consistently identify structural atherosclerotic changes, including increased carotid intima–media thickness (cIMT) and a higher prevalence of carotid plaque. In a meta-analysis of vascular risk markers in PsA, patients exhibited greater cIMT than controls (mean difference 0.07 mm, 95% CI 0.04–0.11) and a markedly increased frequency of carotid plaques (OR 3.12, 95% CI 1.94–5.04), underscoring a substantial burden of subclinical carotid atherosclerosis [17]. Beyond the carotid structure, vascular function is also impaired. In the prospective PSOCARD cohort, which included 150 PsA patients and 88 controls, carotid–femoral pulse wave velocity (cfPWV)—a surrogate marker of arterial stiffness—was significantly higher in PsA even after adjustment for confounders, and 16% of PsA patients exhibited cfPWV values exceeding 10 m/s, a threshold associated with increased aortic stiffness and end-organ involvement [18]. Coronary artery calcium scoring further corroborates an elevated burden of silent coronary atherosclerosis; in a multi-centre cohort using coronary computed tomography angiography, individuals with PsA had significantly greater odds of having a coronary artery calcium score above zero compared with non-inflammatory controls (adjusted OR approximately 1.28) [19]. Collectively, these findings indicate that subclinical CVD is common in PsA and may precede overt cardiovascular events by several years.

## 3. Pathophysiological Links Between PsA and CVD

The heightened cardiovascular risk in PsA is increasingly attributed to immune dysregulation, metabolic disturbance, and chronic systemic inflammation. This interaction is driven by a pro-inflammatory cytokine milieu dominated by tumour necrosis factor-α (TNF-α), IL-17, IL-23, and IL-6, with effects extending beyond the synovio-entheseal complex. Together, these cytokines affect endothelial function, insulin signalling, and lipid metabolism, promoting early atherosclerosis [20,21,22]. Chronic inflammation also promotes a pro-thrombotic state characterized by elevated fibrinogen, impaired fibrinolysis, and increased platelet reactivity [23]. Inflammation-driven autonomic dysregulation, particularly sympathetic activation, may further impair vascular tone and heart rate variability [24]. Understanding the cumulative effect of these disease-driven pathways is crucial for both understanding the increased cardiovascular risk associated with PsA and directing treatment strategies that could lower it.

Innate and adaptive immune responses jointly shape the immunopathogenic link between PsA and CVD [25,26]. Th1 and Th17 cells play a key role in the adaptive compartment, as PsA synovial and cutaneous infiltrates show marked overexpression of mediators such as TNF-α and IL-17, which drive inflammation beyond musculoskeletal tissues. TNF-α promotes insulin resistance, dyslipidaemia, endothelial adhesion molecule expression, and plaque rupture [27,28]. By promoting smooth muscle growth and lipid deposition in the vascular intima and by inducing oxidative stress and endothelial dysfunction, IL-17 further intensifies vascular inflammation. This response is reinforced by the IL-23/IL-17 axis, since IL-23 promotes Th17 differentiation and expansion, increases neutrophil recruitment, and stimulates the production of reactive oxygen species (ROS)—processes associated with endothelial dysfunction, oxidative stress, and myocardial injury [29,30,31]. This inflammatory environment is significantly influenced by innate immunity. By producing ROS and activating inflammasomes, neutrophils and macrophages—which are activated and dysregulated in PsA—infiltrate vascular lesions and actively contribute to the formation of plaque. In synovial and skin tissues, macrophage-derived cytokines and transcriptional mediators, including nuclear factor κB, amplify inflammatory signalling. IL-23-driven myeloid expansion, particularly neutrophilia, further promotes oxidative damage and endothelial dysfunction [30,32,33]. These innate and adaptive immune mechanisms generate a persistent pro-inflammatory, pro-oxidative milieu that drives the metabolic disruptions and accelerated atherogenesis underlying cardiovascular complications in PsA.

In PsA, metabolic abnormalities are common and provide a major pathway linking chronic inflammation to CVD. A clustered cardiometabolic profile, including obesity, insulin resistance, type 2 diabetes, hypertension, dyslipidaemia, and, particularly, metabolic syndrome (MetS), is closely linked to PsA [6,8]. Current consensus definitions describe MetS as a constellation of interrelated risk factors—elevated waist circumference (>102 cm in men, >88 cm in women), elevated blood pressure (systolic > 130 mmHg or diastolic > 85 mmHg), elevated fasting glucose (>100 mg/dL), elevated triglycerides (>150 mg/dL), and reduced high-density lipoprotein cholesterol (<40 mg/dL in men; <50 mg/dL in women)—with the presence of any three fulfilling diagnostic criteria [34]. Compared to the general population and other inflammatory arthropathies, patients with PsA have a significantly higher prevalence of MetS, according to recent meta-analytic data. PsA was associated with roughly 2.5-fold higher odds of MetS than in the general population, 1.9-fold higher odds than in rheumatoid arthritis, and roughly 2.8-fold higher odds than in ankylosing spondylitis in pooled analyses. These findings highlight the contribution of cardiometabolic clustering to cardiovascular risk in PsA [35]. Notably, several meta-analyses have demonstrated that the accumulation of cardiometabolic risk factors, including MetS, is linked to increased global cardiovascular risk and plays a role in the excess cardiovascular morbidity and mortality reported in PsA [12,36]. Thus, MetS and its components interact with PsA-specific inflammatory pathways and amplify cardiovascular burden.

Obesity is a prevalent metabolic disturbance in PsA and a major amplifier of cardiovascular risk. Contemporary cohorts and registry data indicate that the majority of patients with psoriatic disease are overweight or obese, with obesity affecting roughly one-quarter to one-half of individuals with PsA—rates that exceed those observed in the general population and in several other inflammatory arthritides [37]. Obesity in PsA arises from the interplay of chronic systemic inflammation, adipose tissue dysfunction, and reduced physical activity driven by PsA-related pain and disability [38]. Visceral adipose tissue acts as an endocrine organ, promoting adipokine imbalance with increased leptin and resistin and reduced adiponectin. This profile reinforces inflammation, insulin resistance, atherogenic dyslipidaemia, and endothelial dysfunction [39,40]. Clinical studies further indicate that obesity not only increases the likelihood of developing PsA and correlates with higher disease activity and poorer treatment response, but also confers an additive cardiovascular burden when combined with other metabolic risk factors [6,41,42]. Correcting adipokine imbalance through pharmacologic approaches and lifestyle-based weight-reduction strategies may help reduce disease burden and diminish cardiovascular complications in PsA.

Insulin resistance and type 2 diabetes are major contributors to cardiovascular risk in PsA. Current meta-analytic evidence demonstrates that patients with PsA have a significantly increased risk of type 2 diabetes compared with the general population, with pooled analyses indicating approximately a 38% higher incidence of type 2 diabetes in PsA [43]. Beyond being common, diabetes carries important prognostic implications: in a large longitudinal cohort, type 2 diabetes mellitus was independently associated with major cardiovascular events, conferring approximately a 2.7-fold increase in composite cardiovascular risk after multivariable adjustment [4]. More recently, data from the 10-year prospective CARMA cohort demonstrated that a higher metabolic score for insulin resistance identified PsA patients at increased risk of incident cardiovascular events, with an annualized incidence of 5.6 events per 1000 patient-years, supporting insulin resistance as a clinically relevant prognostic marker [44]. Mechanistically, PsA-related inflammation disrupts glucose homeostasis and insulin signalling. TNF-α and IL-6 impair insulin receptor signalling, disrupt downstream Akt activation, and reduce glucose uptake in skeletal muscle and adipose tissue. IL-17 may further exacerbate insulin resistance by promoting adipose tissue inflammation and adipocyte dysfunction. Additionally, obesity-associated adipokine imbalance amplifies this dysregulation by promoting pro-inflammatory signaling and attenuating insulin sensitivity [45,46,47]. These mechanisms promote both diabetes and CVD progression, supporting early metabolic assessment in PsA.

Dyslipidaemia is another major cardiometabolic abnormality contributing to the pro-atherogenic milieu in PsA. In a large meta-analysis of 39 observational studies encompassing over 150,000 PsA patients, approximately 24% of individuals with PsA were estimated to have dyslipidemia [12]. Quantitative lipid abnormalities in PsA include elevated total cholesterol, increased triglycerides, reduced high-density lipoprotein (HDL) cholesterol, and a higher proportion of small, dense low-density lipoprotein (LDL) particles. Qualitative lipid disturbances are also prominent and involve alterations in lipoprotein composition and function, including smaller, denser, and dysfunctional HDL particles, as well as increased production of pro-inflammatory lipid mediators such as leukotriene B4, prostaglandin E1, and thromboxane B2, reflecting enhanced cyclooxygenase-2 activity. These qualitative changes not only promote inflammation and atherogenesis but also modulate immune responses [48,49,50]. Chronic systemic inflammation further disrupts hepatic lipid metabolism by promoting increased synthesis of very-low-density lipoprotein (VLDL) and triglycerides and by enhancing the formation of oxidized LDL. Cytokines such as TNF-α and IL-6 stimulate hepatic de novo fatty acid synthesis and VLDL secretion while suppressing cholesterol elimination pathways, thereby shifting lipid metabolism toward a pro-atherogenic profile [51,52].

Hypertension is often underrecognized in PsA despite its contribution to cardiovascular morbidity and mortality. Meta-analyses indicate that hypertension is common in PsA, with a pooled prevalence of approximately 34%. Furthermore, the odds of hypertension in PsA were roughly 1.5 times higher than those in the general population, according to a meta-analysis spanning immune-mediated diseases [12,53]. Cohort studies link hypertension with cardiovascular events and reduced survival, confirming its prognostic significance in PsA [4,54]. Persistent systemic inflammation is closely associated with the multifactorial development of hypertension in PsA. TNF-α, IL-6, and IL-17 disrupt vascular homeostasis by increasing oxidative stress, reducing nitric oxide bioavailability, and upregulating vasoconstrictors. They may also stimulate the renin–angiotensin–aldosterone system, promoting sodium retention, vascular remodelling, and arterial stiffness. Obesity and insulin resistance further increase blood pressure through sympathetic activation and impaired insulin-mediated vasodilatation [53,55,56].

Although the association between PsA and cardiometabolic abnormalities is consistently reported, interpretation of the available evidence requires consideration of several methodological limitations. Many studies are observational or cross-sectional, limiting causal inference and allowing residual confounding. In addition, heterogeneity in study populations, disease severity, and definitions of metabolic outcomes contributes to variability in reported prevalence estimates across cohorts. Differences in baseline cardiovascular risk, treatment exposure, and lifestyle factors may further influence these associations. Thus, although cardiometabolic clustering in PsA is well supported, the independent contribution of each component requires further prospective study.

**Figure 1:**
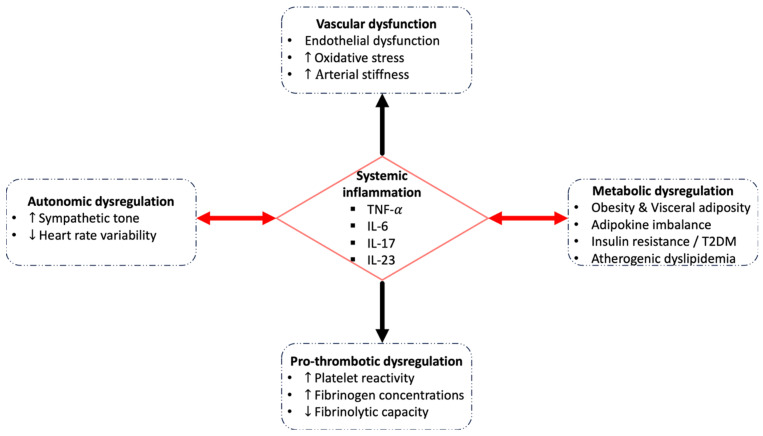
Systemic inflammation represents the central pathophysiological link between psoriatic arthritis (PsA) and cardiovascular disease (CVD).

In summary, PsA and CVD are linked by chronic inflammation, endothelial dysfunction, metabolic disruption, haemostatic imbalance, and autonomic dysregulation, which together increase the risk of MACE and premature mortality (see Figure 1). This multifaceted pathogenic landscape supports integrated strategies to identify and modify both inflammatory and non-inflammatory cardiovascular risk drivers early.

## 4. Impact of PsA Therapies on Cardiovascular Risk

PsA therapies influence not only musculoskeletal and dermatologic outcomes but also cardiovascular risk. Pharmacologic agents may influence cardiovascular risk through two competing pathways: inflammation suppression and drug-specific off-target effects [9,57]. Understanding the net cardiovascular impact of each therapeutic class is therefore crucial when selecting and sequencing treatment in PsA, particularly in patients with established CVD or multiple cardiovascular risk factors [58]. In the following subsections, we review the principal drug classes used in PsA—from nonsteroidal anti-inflammatory drugs (NSAIDs) and glucocorticoids to conventional synthetic, biologic, and targeted synthetic disease-modifying antirheumatic drugs (DMARDs)—with a focus on their mechanisms of action and cardiovascular risk.

NSAIDs are widely used in PsA for symptomatic relief of peripheral arthritis, enthesitis, and axial disease through inhibition of cyclo-oxygenase (COX-1/COX-2) enzymes [59]. However, their cardiovascular safety profile is complex. Meta-analytic data suggest higher cardiovascular risk with COX-2 inhibitors, whereas non-selective NSAIDs show no consistent increase in cardiovascular events [60]. More recent PsA-specific data provide further insight: in a retrospective cohort of approximately 200 PsA patients followed over nearly nine years, use of non-selective NSAIDs was independently associated with a lower incidence of cardiovascular events (HR 0.38), while no significant association was observed for COX-2 inhibitors; however, these findings should be interpreted cautiously, as the observational design limits causal inference [61]. Conversely, recent observational data linking NSAID exposure to cardiovascular comorbidity may partly reflect confounding by indication [62]. NSAIDs remain useful for symptom control but should be used at the lowest effective dose and shortest duration, especially in patients with established CVD or multiple risk factors [58,63].

Systemic glucocorticoids exert significant, broad-spectrum anti-inflammatory effects by binding to intracellular glucocorticoid receptors, modulating gene transcription, and inhibiting various cytokine pathways [64]. Their application in PsA is usually limited to bridging therapy to DMARD escalation or short-term management of severe flares [65]. Glucocorticoids have an unfavourable cardiometabolic profile, promoting central adiposity, insulin resistance, dyslipidaemia, and hypertension [6,66]. A large population-based cohort study showed a dose- and duration-dependent increase in cardiovascular events, such as myocardial infarction and stroke, across immune-mediated inflammatory diseases, with measurable excess risk identified even at daily doses below 5 mg prednisolone-equivalent [67]. PsA-specific real-world data similarly associate current glucocorticoid use with greater cardiovascular comorbidity burden [62]. These findings are supported by the updated European Alliance of Associations for Rheumatology (EULAR) guidelines for PsA, which recommend limiting systemic glucocorticoid use to short courses, especially in patients with multiple cardiometabolic risk factors or established CVD [58].

In PsA management, conventional synthetic DMARDs—mainly methotrexate (MTX), leflunomide, and sulfasalazine—remain essential. Through different molecular mechanisms, these agents modify disease: MTX suppresses downstream purine metabolism and folate-dependent enzymatic activity; leflunomide reduces T-cell activation by preventing dihydroorotate dehydrogenase-mediated pyrimidine synthesis; and sulfasalazine regulates the release of inflammatory mediators and oxidative stress [68,69,70]. MTX has the strongest evidence of cardiovascular benefit among conventional synthetic DMARDs. MTX use is associated with lower cardiovascular and all-cause mortality, with effect estimates of about 20–30% lower risk of major cardiovascular events, according to meta-analyses of observational cohorts in rheumatoid and related inflammatory arthritis [71,72,73]. Leflunomide appears less favourable from a cardiovascular perspective. Data from other inflammatory diseases suggest possible blood pressure elevation, warranting monitoring in patients with cardiovascular risk [74]. Sulfasalazine appears largely cardiovascularly neutral. Available data do not suggest increased MACE, although PsA-specific outcome studies are lacking [75,76,77]. These differences support considering cardiovascular profile alongside therapeutic efficacy.

Tumor necrosis factor inhibitors (TNFi) block TNF-α–mediated inflammatory signaling and represent a cornerstone of PsA management [33]. TNFi have the most consistent cardiovascular evidence among biologics, with observational and meta-analytic data suggesting lower MACE rates and improved vascular markers. While PsA-specific datasets remain more limited, the available studies show comparable trends toward cardiovascular benefit [78,79,80,81]. Interleukin-17 and interleukin-23 inhibitors (IL-17i/IL-23i) block key cytokine pathways within the IL-23/IL-17 axis, a principal driver of psoriatic immune dysregulation [82]. Contemporary cardiovascular safety data for IL-17 and IL-23 inhibitors in psoriatic disease are generally reassuring, although still maturing. A recent meta-analysis found no significant increase in MACE with IL-17 inhibition versus placebo, supporting a neutral cardiovascular profile [83]. Population-based data also suggest lower CVD event rates with IL-17 or IL-23 inhibitors than with conventional systemic therapies. However, absolute event numbers remain limited, follow-up duration is modest, and observational design precludes causal inference [84]. As a result, while current evidence supports a reassuring—potentially favorable—cardiovascular profile for IL-17 and IL-23 inhibition, definitive conclusions regarding differential cardiovascular effects between the two classes cannot yet be drawn, particularly in PsA-specific cohorts.

Targeted synthetic DMARDs used in PsA primarily include JAK inhibitors, such as tofacitinib and upadacitinib, and the phosphodiesterase-4 (PDE4) inhibitor apremilast. JAK inhibitors modulate intracellular inflammatory signalling by blocking JAK–STAT pathways downstream of multiple cytokine receptors [85]. However, cardiovascular safety concerns have emerged: in the ORAL Surveillance trial in rheumatoid arthritis, tofacitinib was associated with increased rates of MACE and venous thromboembolism compared with TNFi in patients aged ≥50 years with pre-existing cardiovascular risk [86]. Subsequent regulatory assessments extended these safety warnings to all approved JAK inhibitors used in chronic inflammatory disease. PsA-specific real-world data show low absolute event numbers but support caution in patients with elevated baseline cardiovascular risk [87,88]. Apremilast, a selective PDE4 inhibitor, increases intracellular cAMP and downregulates pro-inflammatory cytokines such as TNF-α and IL-23 [89]. Unlike JAK inhibitors, apremilast has not been associated with increased MACE in large real-world psoriasis and PsA cohorts [90,91]. Beyond safety, accumulating data suggest potentially favourable cardiometabolic effects: observational studies in psoriatic disease report modest weight loss—often driven by reductions in abdominal fat—together with improvements in lipid parameters [92,93]. Overall, apremilast appears cardiovascularly neutral and potentially metabolically favourable.

Despite growing interest in the cardiovascular implications of PsA therapies, the current evidence base remains subject to several important limitations. Much of the evidence comes from observational cohorts or meta-analyses with heterogeneous inflammatory disease populations, limiting PsA-specific inference. Confounding by indication is also likely, as treatment groups often differ in disease severity, inflammatory burden, and baseline cardiovascular risk. Moreover, PsA trials usually target musculoskeletal and dermatologic outcomes rather than cardiovascular endpoints. Consequently, although several therapeutic classes appear to have favourable or neutral cardiovascular profiles, definitive conclusions regarding their long-term cardiovascular effects in PsA remain limited.

PsA therapies have heterogeneous cardiovascular implications (see Table 1). Long-term cardiovascular trajectories are as much influenced by therapeutic choice as musculoskeletal outcomes; some medications reduce risk by efficiently reducing systemic inflammation, while others may increase vulnerability by affecting metabolism or haemodynamics. Thus, it is imperative—rather than optional—to incorporate cardiovascular profiles into routine treatment decisions. This ensures that cardiometabolic safety and disease-modifying efficacy are evaluated simultaneously to maximise results in both areas.

## 5. Cardiovascular Risk Assessment and Screening in PsA

The poor estimation of cardiovascular risk in PsA by general-population algorithms without disease-specific adjustment has increasingly been acknowledged. Ernste et al. reported significantly more cardiovascular events than anticipated, but early cohort work in newly diagnosed PsA showed that the Framingham Risk Score (FRS) significantly underestimated observed cardiovascular events. This suggests that accelerated vascular risk appears early and cannot be fully explained by traditional factors alone [94]. Additional observational and imaging studies demonstrate that instruments like the FRS, Systematic Coronary Risk Evaluation (SCORE), QRISK, and Reynolds Risk Score (RRS) frequently exhibit poorer calibration and discrimination; multiple groups have reported that these scores may overlook subclinical carotid atherosclerosis as well as clinical events, even in patients with low or intermediate risk [95]. External validation studies confirm poorer performance in PsA than in non-inflammatory controls. Miscalibration may involve either overestimation or underestimation, indicating algorithm-specific rather than uniform bias [96,97]. All of the data point to the need for better, disease-adjusted CV risk stratification, as existing general-population algorithms are insufficient for capturing the inflammation-related vascular burden of PsA.

**Figure 2:**
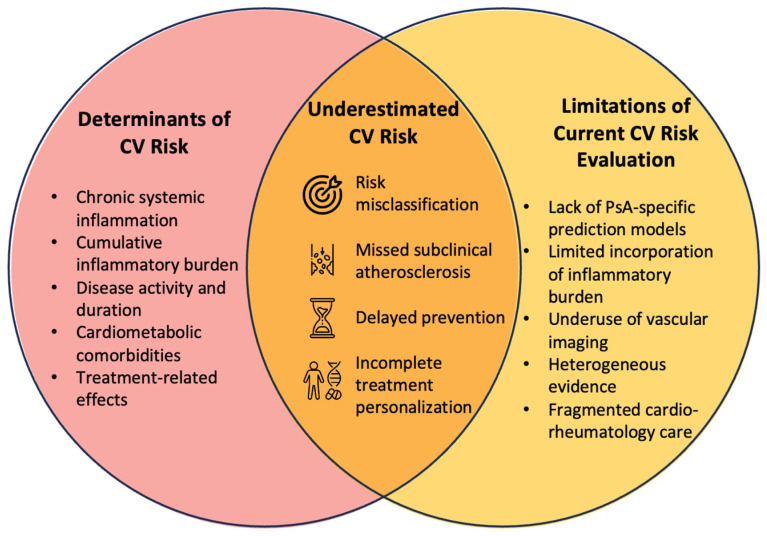
Determinants and limitations contributing to underestimated cardiovascular (CV) risk in PsA.

In recognition of this limitation, rheumatology societies have advocated adjusting traditional risk scores using an inflammatory “multiplier” approach derived from data on rheumatoid arthritis (RA). The original and updated EULAR recommendations for cardiovascular risk management in inflammatory joint disorders propose that, in RA, calculated CV risk should be multiplied by a factor of 1.5 when certain disease-related criteria are met—long disease duration, seropositivity, or severe extra-articular manifestations—to approximate the excess risk attributable to chronic inflammation [98]. Although this multiplier was established from RA cohorts and remains empiric, comparative studies show that PsA and spondyloarthritis exhibit similarly increased cardiovascular risk, supporting why some clinicians have extrapolated this strategy to PsA in the absence of disease-specific algorithms [99]. However, in an Italian bicentric PsA cohort, applying the 1.5 multiplier to SCORE, Framingham, CUORE, QRISK2, and RRS did not improve discrimination or calibration, and many future event-positive patients remained categorized as low- or intermediate risk—highlighting the poor performance of this adjustment in PsA [95]. Accordingly, EULAR endorses the multiplier for RA, but not for PsA [58]. Commonly used tools include SCORE2, the ASCVD risk estimator, Framingham-derived scores, and QRISK models. These tools differ in their derivation cohorts, predicted outcomes, and regional calibration. For example, SCORE2 and SCORE2-OP were developed for European populations and estimate the 10-year risk of fatal and non-fatal cardiovascular events, whereas the ASCVD risk estimator is based on pooled cohort equations derived from U.S. populations and predicts the probability of a first atherosclerotic cardiovascular event. Other models, including Framingham-derived scores and QRISK algorithms, incorporate different combinations and weighting of traditional cardiometabolic risk factors. Despite these differences, all rely mainly on conventional cardiovascular variables. Psoriatic disease-specific models suggest that traditional factors remain dominant, but disease duration, activity, and systemic inflammation may refine risk stratification [100]. These findings highlight the limitations of applying general-population models to inflammatory diseases. Conventional models omit disease-specific determinants such as systemic inflammation, cumulative inflammatory burden, and treatment effects, which may accelerate atherosclerosis in PsA. As a result, they may underestimate risk in chronic inflammatory disorders (see Figure 2). Integrating inflammatory disease characteristics may improve PsA risk prediction, but prospective validation is needed before routine use. Until PsA-specific models are validated, conventional scores should be interpreted conservatively, especially in long-standing, active, or severe PsA.

By directly visualising subclinical atherosclerosis and improving risk reclassification in PsA, non-invasive vascular imaging has evolved into a useful asset for clinical calculators. Meta-analytic data show higher cIMT and carotid plaque prevalence in PsA than in matched controls [17]. Similarly, coronary artery calcium (CAC) scoring, which offers additional prognostic information beyond clinical scores alone, detects an elevated coronary calcific burden in psoriatic populations [101]. In light of these findings, recent critical reviews of cardiovascular imaging in psoriasis and PsA recommend that carotid ultrasound and CAC scoring be utilised selectively for risk refinement in asymptomatic patients, specifically those with multiple risk factors or high cumulative inflammatory exposure, while preserving more advanced modalities (coronary CT angiography) for patients with suggestive symptoms or abnormal screening results [102,103].

In this context, cardiovascular risk screening in PsA must evolve from intermittent laboratory monitoring to a systematic, inflammation-sensitive evaluation framework that acknowledges its established high-risk status. EULAR guidance supports systematic assessment, identifying metabolic syndrome and CVD as priority comorbidities in psoriatic disease [58]. According to the Psoriasis & Psoriatic Arthritis Clinics Multicenter Advancement Network guidelines, all patients with psoriasis and PsA—especially those with established PsA, moderate-to-severe inflammatory burden, or biologic therapy—should have a formal cardiovascular evaluation at the time of diagnosis. This evaluation should include a systematic assessment of hypertension, dyslipidemia, insulin resistance/type 2 diabetes, obesity, smoking status, and metabolic syndrome as principal determinants of short and long-term risk [103]. The European Society of Cardiology’s (ESC) CVD prevention strategies support population-based risk stratification at roughly five-year intervals in the general adult population and more frequent reassessment in individuals with heightened clinical vulnerability. These strategies emphasize repeated cardiovascular assessment rather than a one-time calculation.

At the very least, routine screening should include standardised blood pressure measurement, home or ambulatory monitoring if white-coat or masked hypertension is suspected, a full lipid profile, and tests for impaired glucose metabolism using fasting glucose and HbA1c. If necessary, renal function tests should also be done. Anthropometric assessment should include both body mass index and waist circumference. In individuals with long-standing inflammatory disease, numerous cardiometabolic risk factors, or borderline/intermediate calculated risk, adjunctive imaging can refine risk stratification: ESC prevention guidelines acknowledge coronary artery calcium scoring as a validated modifier of clinical risk, with carotid ultrasound recognised as a recommended alternative for detecting subclinical atherosclerosis and directing the intensity of preventive intervention [104,105].

**Figure 3:**
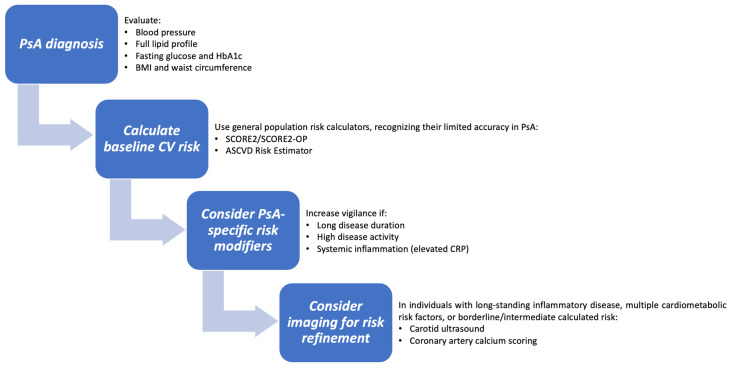
Proposed algorithm for CV risk assessment in patients with PsA.

In conclusion, improving cardiovascular risk assessment in PsA requires three changes to occur simultaneously: recognising that traditional risk calculators do not adequately account for risk; adding imaging and inflammatory markers to risk stratification; and adopting a proactive, rather than reactive, screening approach (see Figure 3). Together, these measures form the basis of preventive cardiovascular care in PsA.

## 6. Integrated Management: A Cardio-Rheumatology Care Model

Optimal management of cardiovascular risk in PsA extends beyond isolated specialty care and requires a deliberately coordinated, interdisciplinary model. The emerging cardio-rheumatology framework embodies this shift, integrating disease-modifying antirheumatic therapy and cardiovascular risk reduction into a single, unified clinical strategy rather than parallel, disconnected efforts. At its core lies a fundamental recognition: in PsA, rigorous control of systemic inflammation is not merely a rheumatologic objective but a cardiovascular intervention in its own right [78]. Within this cardio-rheumatology framework, management centres on four tightly interlinked domains: shared therapeutic decision-making, harmonised treatment targets, structured lifestyle and weight management, and pharmacotherapy.

Shared decision-making implies that the cardiovascular profile is now explicitly used to guide the selection and sequencing of PsA therapies, rather than solely on articular or cutaneous efficacy. Current EULAR guidelines already highlight comorbidity-driven pharmacologic therapy tailoring, advising against long-term systemic glucocorticoid use, recommending MTX as a first-line csDMARD, and warning against JAK inhibitors in patients with multiple cardiovascular risk factors or established atherosclerotic disease [58]. Concurrently, growing observational and meta-analytic data on psoriatic disease and associated spondyloarthropathies indicate that a decrease in major adverse cardiovascular events is associated with effective TNFi suppression of inflammation, whereas the cardiovascular impact of more recent biologics appears largely neutral [78]. Rheumatologists and cardiologists discuss these different profiles together within an integrated model. The goal is to choose regimens that keep PsA activity under control while either lowering or, at the very least, not raising long-term cardiovascular risk.

Harmonised treatment targets represent the second axis of integrated management. Treat-to-target strategies in PsA, using composite indices such as Disease Activity Index for Psoriatic Arthritis (DAPSA) or Psoriatic Arthritis Disease Activity Score (PASDAS), must be aligned with guideline-directed cardiometabolic goals derived from contemporary ESC prevention frameworks—risk-stratified LDL-cholesterol thresholds, strict blood pressure control, and individualised glycaemic targets in patients with diabetes or pre-diabetes [104,105,106]. In this context, achieving remission or low disease activity and attaining lipid, blood pressure, and glucose targets should be conceptualised as parallel, co-primary priorities rather than independent therapeutic tasks. Accordingly, treatment intensification should be considered when either inflammatory or cardiometabolic goals remain unmet [78].

The third essential pillar of integrated care is lifestyle and weight management. In PsA, obesity is significantly over-represented and is consistently associated with increased cardiovascular risk, decreased responsiveness to treatment, and increased disease activity. On the other hand, it has been demonstrated that even a small, deliberate weight loss, such as 5% of baseline body weight, can improve treatment responsiveness, including to TNFi, and increase the likelihood of achieving minimal disease activity in patients who are overweight or obese [37]. In line with these findings, EULAR lifestyle guidelines recommend professional, structured interventions that emphasise physical conditioning, visceral adiposity reduction, and customised nutrition, ideally carried out in a multidisciplinary setting [107].

Finally, pharmacotherapy functions as a core intervention within integrated management, complementing lifestyle measures and consolidating cardiovascular risk reduction. Statins and renin–angiotensin system blockers remain the cornerstone of lipid and blood pressure management, respectively, providing robust reductions in atherosclerotic events and target-organ damage when deployed according to contemporary prevention guidelines [104,105]. Building on this foundation, sodium–glucose cotransporter 2 (SGLT2) inhibitors and glucagon-like peptide-1 (GLP-1) receptor agonists have broadened the therapeutic armamentarium, demonstrating significant reductions in heart failure hospitalisations, cardiovascular mortality, and adverse renal outcomes in high-risk cardiovascular populations, including individuals without diabetes [108]. A recent meta-analysis further shows that GLP-1 receptor agonists not only promote sustained weight loss but also reduce major adverse cardiovascular events in patients with type 2 diabetes and, more recently, in people with obesity but no diabetes [109]. These findings position GLP-1 receptor agonists and SGLT2 inhibitors as particularly compelling options for PsA patients with concomitant obesity, diabetes, or markedly elevated global cardiovascular risk. In parallel, low-dose aspirin continues to have a clearly defined role in secondary prevention of atherosclerotic CVD, while its use in primary prevention is increasingly restricted to carefully selected high-risk individuals [104,105].

**Figure 4:**
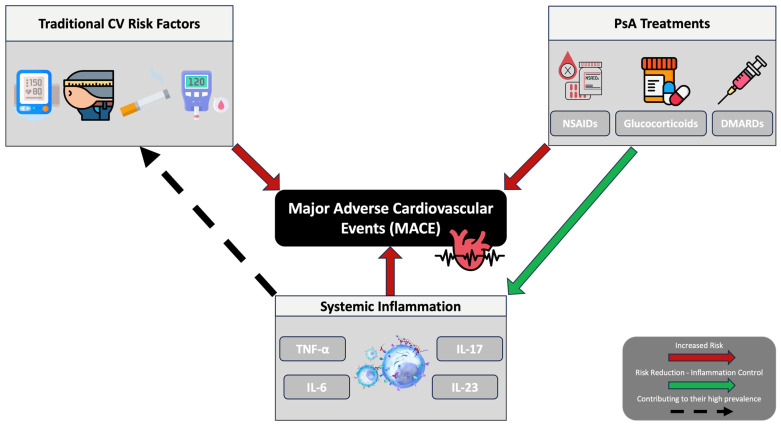
Complex interplay between traditional cardiovascular risk factors, systemic inflammation, PsA treatments, and major adverse cardiovascular events.

Therefore, cardiovascular risk management in PsA must be integrated directly into the therapeutic strategy rather than addressed as a peripheral concern. A cardio-rheumatology model operationalises this approach by aligning treatment selection with the patient’s cardiovascular profile, pursuing inflammatory and cardiometabolic endpoints as co-primary targets, embedding structured lifestyle intervention, and initiating pharmacotherapy with demonstrable cardiovascular benefit. The complex interaction between systemic inflammation, cardiometabolic abnormalities, therapeutic effects, and cardiovascular outcomes in PsA is summarized in the conceptual model presented in Figure 4.

## 7. Unmet Needs and Future Directions

Cardiovascular risk prediction in PsA remains limited by the absence of validated disease-specific tools. Commonly used general-population algorithms consistently perform worse in psoriatic disease than in the cohorts from which they were derived, according to external validation studies. Emerging PsA/psoriatic disease-specific models that combine traditional risk factors with disease-related measures are promising, but remain insufficiently validated for routine clinical use [100]. Artificial intelligence may further refine risk stratification by integrating multidimensional clinical, inflammatory, metabolic, and imaging data. While recent reviews of psoriatic disease describe how AI-enabled models could support risk prediction and clinical decision-making, early work has already shown that machine learning-based cardiovascular risk prediction in PsA is feasible [78,110].

Randomised cardiovascular endpoint-driven trials in psoriatic disease remain scarce. Much of the current data linking PsA treatments to cardiovascular outcomes is derived from observational cohorts and meta-analyses, which are useful but remain susceptible to indication bias and residual confounding [111]. Prospective studies with predefined cardiovascular endpoints are needed to clarify treatment-related cardiovascular effects and guide risk-informed therapeutic decision-making.

Preventive strategies should focus on earlier stages of psoriatic disease. Based on imaging and biomarker studies, subclinical atherosclerosis is common in PsA and can occur years before overt cardiovascular events. Prevention during this early phase may be more effective than late-stage risk modification [112]. To enable preventive intervention during a window of maximum reversibility, future strategies should focus on identifying high-risk individuals at earlier stages, possibly through the combined use of inflammatory biomarkers, adipokine profiles, and non-invasive vascular imaging.

Integrated cardio-rheumatology care remains inconsistently implemented. Even though their importance is increasingly recognized, these models are still not consistently incorporated into standard clinical practice [113]. Implementation requires defined screening intervals, referral criteria, shared inflammatory and cardiometabolic targets, and clear coordination between rheumatology, cardiology, primary care, and metabolic specialists.

Looking forward, future developments in precision medicine could improve cardiovascular prevention in PsA. Individualised cardiovascular risk profiling may be achieved by combining multi-omics data with AI-driven risk prediction. These approaches could eventually enable tailoring anti-inflammatory and cardioprotective treatments to the inflammatory and metabolic signatures unique to each patient, surpassing population-based thresholds and accomplishing truly personalised prevention.

## 8. Conclusions

PsA should be reframed as a systemic inflammatory disease, in which CVD is a significant, potentially preventable factor influencing long-term results. Prothrombotic dysregulation, endothelial dysfunction, cytokine-mediated vascular inflammation, and inflammation-induced metabolic abnormalities all interact in an intricate manner to accelerate atherogenesis and increase cardiovascular vulnerability over the course of the disease, contributing to the excess burden of major adverse cardiovascular events that extends beyond the mere accumulation of traditional risk factors. The suppression of inflammation is a cardiovascular intervention, but medication selection must account for baseline risk and cardiometabolic trade-offs. This pathobiological convergence has direct therapeutic implications. Evidence currently available also calls for a shift in practice, moving away from episodic screening toward structured, longitudinal risk assessment that recognises the limitations of general-population calculators. Ultimately, optimal care requires a cardio-rheumatology model in which treat-to-target control of PsA activity and guideline-directed cardiometabolic prevention goals are pursued as co-primary objectives. Progress now depends on filling in the gaps in risk stratification and prevention techniques, possibly with the support of artificial intelligence, to achieve precision prevention at scale.

## Figures and Tables

**Table 1:** Cardiovascular effects of psoriatic arthritis therapies.

Therapy Class	Mechanism of Action	Reported CV Effect	Key Evidence
NSAIDs	COX inhibition	Possible ↑ CV risk, especially with COX-2 inhibitors	Meta-analysis and observational studies
Glucocorticoids	Glucocorticoid receptor-mediated cytokine suppression	↑ CV risk via adverse metabolic effects	Cohort studies
MTX	Inhibition of purine and folate-dependent pathways	Possible ↓ CV events	Meta-analyses
Leflunomide	Dihydroorotate dehydrogenase inhibition	Possible blood pressure elevation	Observational studies
Sulfasalazine	Modulation of inflammatory mediators and oxidative stress	Neutral CV profile	Observational data
TNF inhibitors	TNF-α signalling blockade	↓ MACE risk	Meta-analyses
IL-17 inhibitors	IL-17 pathway inhibition	Neutral CV profile	Meta-analysis
IL-23 inhibitors	IL-23 pathway inhibition	Reassuring safety; limited long-term data	Cohort studies
JAK inhibitors	JAK-STAT signalling inhibition	↑ MACE risk in high-risk patients	Randomized safety trial and real-world data
Apremilast	PDE4 inhibition	Neutral CV profile; possible metabolic benefit	Real-world cohorts and observational studies

## Data Availability

No new data were created or analyzed in this study. Data sharing is not applicable to this article.

## Abbreviations

The following abbreviations are used in this manuscript:

PsA	psoriatic arthritis
MACE	major adverse cardiovascular events
TNF-α	tumour necrosis factor-α
JAK	Janus kinase
CVD	cardiovascular disease
RR	relative risk
cIMT	carotid intima–media thickness
cfPWV	carotid–femoral pulse wave velocity
IL-17	interleukin-17
IL-23	interleukin-23
IL-6	interleukin-6
ROS	reactive oxygen species
MetS	metabolic syndrome
HDL	high-density lipoprotein
LDL	low-density lipoprotein
VLDL	very-low-density lipoprotein
NSAIDs	nonsteroidal anti-inflammatory drugs
DMARDs	disease-modifying antirheumatic drugs
COX	cyclo-oxygenase
EULAR	European Alliance of Associations for Rheumatology
MTX	methotrexate
TNFi	Tumor necrosis factor inhibitors
PDE4	phosphodiesterase-4
FRS	Framingham Risk Score
SCORE	Systematic Coronary Risk Evaluation
RRS	Reynolds Risk Score
RA	rheumatoid arthritis
CAC	coronary artery calcium
ESC	European Society of Cardiology
DAPSA	Disease Activity Index for Psoriatic Arthritis
PASDAS	Psoriatic Arthritis Disease Activity Score
SGLT2	sodium–glucose cotransporter 2
GLP-1	glucagon-like peptide-1
